# Role of the H1 haplotype of microtubule-associated protein tau (*MAPT*) gene in Greek patients with Parkinson's disease

**DOI:** 10.1186/1471-2377-9-26

**Published:** 2009-06-28

**Authors:** Nikolaos Refenes, Juliane Bolbrinker, Georgios Tagaris, Antonio Orlacchio, Nikolaos Drakoulis, Reinhold Kreutz

**Affiliations:** 1 School of Pharmacy, Department of Pharmaceutical Technology, National and Kapodistrian University of Athens, Athens, Greece; 2 Institute of Clinical Pharmacology and Toxicology, Charité – Universitätsmedizin Berlin, Berlin, Germany; 3 Department of Neurology, "G. Gennimatas" General Hospital, Athens, Greece; 4 Laboratory of Neurogenetics, CERC-IRCCS Santa Lucia, Rome, Italy; 5 Department of Neuroscience, University of Rome "Tor Vergata", Rome, Italy

## Abstract

**Background:**

The extended tau haplotype (H1) that covers the entire human microtubule-associated protein tau (*MAPT*) gene has been implicated in Parkinson's disease (PD). Nevertheless, controversial results, such as two studies in Greek populations with opposite effects, have been reported. Therefore, we set out to determine whether the H1 haplotype and additional single nucleotide polymorphisms (SNPs) included in H1 are associated with PD in a sample of Greek patients.

**Methods:**

We analysed *MAPT *haplotypes in cohorts of 122 patients and 123 controls of Greek origin, respectively. SNP genotyping was performed with Taqman assays and genotyping results were confirmed by sequencing.

**Results:**

The presence of the H1 haplotype was significantly associated with PD (odds ratio for H1H1 vs. H1H2 and H2H2: 1.566; 95% CI: 1.137–2.157; *P *= 0.006) and remained so after adjustment for sex. Further analysis of H1 sub-haplotypes with three single nucleotide polymorphisms (rs242562, rs2435207 and rs3785883) demonstrated no significant association with PD.

**Conclusion:**

Our data support the overall genetic role of *MAPT *and the H1 haplotype for PD susceptibility in Greek patients. However, the previously supported association of H1 sub-haplotypes with PD could not be confirmed in our study.

## Background

Parkinson's disease (PD) is the most common movement disorder that becomes more prevalent with advanced age and represents the second most common neurodegenerative disorder after Alzheimer's disease (AD) [1]. PD is characterized by four cardinal symptoms: resting tremor, bradykinesia, muscle rigidity and postural instability [2]. The degeneration of the nigrostriatal dopaminergic neurons causes symptoms of PD and one of the main neuropathological features of the disease consists of intracellular proteinaceous inclusions called Lewy bodies [3]. Aggregation and fibrillization of the α-synuclein protein, which is the main component of Lewy bodies, represent key events in the pathogenesis of PD,[4] and the disease is classified as an α-synucleinopathy.

In addition, a disease mechanism based on the protein tau has been proposed in PD [5,6]. Tau proteins are a group of phosphorylated neuronal microtubule-associated proteins that bind to microtubules and promote microtubule assembly and stabilization. They are expressed in neurons and they are particularly abundant in axons [7]. Due to the proposed interactions of α-synuclein and tau protein and their abnormal intracellular aggregation in neurodegenerative diseases,[5,6] the analysis of microtubule-associated protein tau (*MAPT*) gene as a genetic susceptibility factor for PD has been of interest.

The *MAPT *gene is encoded on chromosome 17q21 in the centre of a 900 kb fragment between two extended haplotypes, H1 and H2, which cover the entire *MAPT *gene [8]. H1 and H2 haplotypes differ in orientation,[9] and do not recombine [10]. Chromosome 17q, containing the *MAPT *gene, was one of the regions given the highest logarithm of odds (lod) scores in the genomic screen for PD conducted by Scott et al [11]. The H1 haplotype of the *MAPT *gene had already been associated with the pathogenesis of parkinsonism tauopathies as progressive supranuclear palsy and corticobasal degeneration [12]. Subsequently, the question arose whether H1 homozygosity would be associated with an increased risk of PD as well. So far, studies, [13-26] have mostly observed an increase in the frequency of the H1H1 genotype in patients with PD but they did not always reach levels of statistical significance (for review see Zabetian et al., 2007) [27].

Originally, genetic analysis in *MAPT *was done by differentiating between H1 and H2 haplotypes. This has been done by analyzing an intron 9 insertion/deletion polymorphism, with the 238 bp deletion being characteristic of the H2 haplotype [8]. The H1 haplotype is more prevalent in Caucasians [28]. Therefore, sub-haplotype analysis for H1 carriers has been conducted by investigating several SNPs [16,17,25,27,29-31]. A positive association between H1 sub-haplotypes and PD was first reported in Norwegian patients,[30] involving SNPs rs242562 (G/A) and rs2435207 (G/A). The 'A-A' sub-haplotype for these SNPs was significantly associated with PD in this Norwegian cohort, while the 'G-A' sub-haplotype for the same SNPs was significantly represented in cases compared to controls in a Greek study [16]. However, in another independent study in Greek patients a moderate association with PD was identified for a different SNP, namely rs3785883 [17].

Since the two previous studies in Greek PD patients gave conflicting results, [16,17] we sought to provide more information on whether the H1 haplotype and H1 sub-haplotypes are associated with PD in this ethnic group of patients. We tested a cohort of PD patients and controls from a different site (Athens) of Greece than the previous two studies which were based on samples from Northern and Central Greece.

## Methods

### Subjects

We recruited 122 unrelated sporadic PD patients (mean age: 64.5 ± 10.7 years, 41% female, mean age of diagnosis: 51.5 years). The patients were of Greek ancestry and were selected by G.T. from the Department of Neurology, "G. Gennimatas" General Hospital, Athens, Greece. All patients had idiopathic PD and did not suffer from other neurological diseases. The process of sample collection did not include any intervention that is not part of any common clinical practice. Idiopathic Parkinson's disease (PD) was diagnosed according to the criteria of the UK Parkinson's Disease Society Brain Bank. The use of the UKPDS standard diagnostic criteria has been shown to increase diagnostic accuracy reaching levels of up to 90% [32]. The PD symptoms were quantified by applying Part III of the Unified Parkinson's Disease Rating Scale (UPDRS) [33] score. The control group consisted of 123 unrelated individuals (mean age: 63.7 ± 17.2, 33.3% female) who were of Greek ancestry as well. The control subjects donated blood during their treatment inAthens Trauma Hospital KAT, and in the Onassis Cardiac Surgery Center, Athens, Greece. The ethics review committees of the hospitals approved the study and written informed consent was obtained from all subjects.

### Selection of polymorphisms

Our primary objective was to select *MAPT *polymorphisms which were previously suggested to contribute to the risk of developing idiopathic PD in Greek subjects. Therefore, we focussed on the H1/H2 insertion/deletion polymorphism, rs242562, rs2435207, and rs3785883 and their sub-haplotypes, which were associated with PD in Greek patients [16,17]. Furthermore, we aimed to provide more data on *MAPT *variants with a potential functional role on the regulation of *MAPT *in tauopathies, since increased expression of H1 haplotype has been suggested as a mechanism of PD susceptibility [20]. SNP rs242557 has been implicated as a functional variant affecting transcriptional activity of tau in patients with Alzheimer's disease [34].

The SNP rs242557 was reported to be strongly associated with rs242562 (r^2 ^= 0.96) by Zabetian et al. [27]. Nevertheless, taking into account its potential functional role we analyzed rs242557 in our study. It turned out that rs242557 was in complete LD with rs242562 among our H1H1 subgroup of patients. The latter SNP is included in a three-locus clade containing also rs 3785883 and rs2471738, which has been strongly associated with the tauopathies in genetic studies [35] and in a quantitive trait analysis in Alzheimer's disease [34]. The strongest two-locus haplotype identified having a significant effect on tau levels in the Alzheimer's disease study was the one containing both rs242557 and rs 3785883.

Overall, we have analyzed 5 polymorphisms at *MAPT *including the H1/H2 polymorphism, rs242562, rs2435207, rs242557, and rs3785883. We did not genotype rs2471738 which is also included in the three-locus haplotype analysis previously referred, because it is known that this SNP is in strong LD with rs2435207 according to the study by Zabetian et al. who reported D' = 0.98 in PD patients and D' = 0.97 in controls [27]. Taken together we were able to represent the previously referred two and three-locus functional sub-haplotypes by the already selected SNPs.

### Genetic analysis of H1 and H2 haplotype

Blood samples were drawn for DNA extraction, using the QIAamp DNA Blood Mini Kit (Qiagen GmbH, Hilden, Germany) and following the manufacturer's protocol, from patients and controls.

The H1/H2 haplotype differentiation was based on the insertion/deletion polymorphism and has been performed as reported by Baker et al.,[8] with minor modifications (Table 1).

**Table 1:** PCR-conditions and sequences of H1-SNP-specific primers.

Primer	Sequences	PCR-conditions
H1 F H1 R	5'-GGA AGA CGT TCT CAC TGA TCT G-3' 5'-AGG AGT CTG GCT TCA GTC TCT C-3'	95°C 5 min, 35× (95°C 30s, 55°C 30s, 72°C 30s), 72°C 10 min
rs242562 F rs242562 R	5'-GGC GAT TCC GCT GAG TCA CC-3' 5'-GGC CCT GCT GGA GTC AAG AG-3'	95°C 5 min, 35× (95°C 30s, 62°C 30s, 72°C 30s), 72°C 10 min
rs2435207 F rs2435207 R	5'-CTG AGG GCC GTC ACT GTC TG-3' 5'-CCT CAA GCC CAT TCT CTG AC-3'	95°C 5 min, 35× (95°C 30s, 58°C 30s, 72°C 30s), 72°C 10 min

F, forward; R, reverse; SNP, single-nucleotide polymorphisms.

Further genetic analysis in H1H1 carriers was performed by genetic determination of rs242562, rs2435207, rs3785883 and rs242557. The ABI Prism^® ^7000 SDS instrument in conjunction with the ABI TaqMan Universal Master Mix was used to perform the assays obtained from Applied Biosystems (Applied Biosystems, Foster City, CA, USA). Data were analyzed using the ABI Prism^® ^7000 SDS 1.0 Software (Perkin-Elmer, Applied Biosystems Division). Correctness of genotyping results was confirmed by sequencing on an ABI 3100 automatic sequencer using the Big-Dye Terminator Cycle Sequencing Ready Reaction Kit (Applied Biosystems, Foster City, CA, USA), for SNPs rs242562 and rs2435207. Therefore, specific fragments involving rs242562 and rs2435207 were amplified by PCR (Table 1). Prior to sequencing, reaction mixtures were purified with a PCR Purification Kit (Qiagen GmbH, Hilden, Germany). For SNP rs3785883 accuracy of genotypes was evaluated by repeated genotyping of one half of the samples.

### Statistical analysis

A χ^2^-test was used to compare the allele frequency of each variant with that expected for a population in Hardy-Weinberg equilibrium. Fisher's exact test was used to compare H1H1, H1H2, and H2H2 genotype distribution between cases and control.

Odds ratios (OR) were calculated with 95% confidence intervals (CI) and exact two-sided *P*-values, using the SPSS 15.0 program for Windows. Logistic regression analysis was conducted to adjust for sex differences. The same statistical procedure was used for genetic analysis of SNPs rs242562, rs2435207 and rs3785883. In addition, the square of the correlation coefficient (R^2^) and D' linkage disequilibrium (LD) was calculated pairwise between H1-SNPs in cases and control subjects, using Haploview 4.1 [36]. The PHASE 2.0 software [37] was used for estimating the frequencies of the sub-haplotypes in H1 homozygous patients and controls. *P*-values were considered significant at *P *< 0.05.

## Results

### H1 and H2 haplotype analysis

The *MAPT *genotype distribution in PD patients and controls is summarized in Table 2. The observed frequencies do not deviate from those predicted by Hardy-Weinberg equilibrium (Table 2) and were comparable to those previously reported in Caucasians [28]. The H1H1 genotype was significantly associated with PD (OR for H1H1 vs. H1H2 and H2H2: 1.566; 95% CI: 1.137–2.157; *P *= 0.006). After adjustment for sex, the strong association with H1H1 remained (OR for H1H1 vs. H1H2 and H2H2: 2.105; 95% CI: 1.250–3.546; *P *= 0.005). Subsequently, we determined H1 sub-haplotypes in individuals carrying the H1H1 genotype, in the cohort of patients (n = 84) and controls (n = 63) of our study, respectively. These results are summarized in Table 3. SNP rs242557 was in complete LD with rs242562 among our H1H1 subgroup of patients, therefore this SNP was discarded from further results analysis. The genotype and allelic distributions were also in Hardy-Weinberg equilibrium for PD patients and controls. Fisher's exact test *P*-values did not reveal any association between the rs242562, rs2435207, rs3785883 SNPs and PD. LD (calculated as D' and R^2^) among H1-SNPs were similar in cases and controls, with the exception of D' = 0.76 between rs3785883 and rs 2435207 in cases, which was also greater than the D' reported in previous studies [25,27] between these two SNPs (Table 4). We also examined the distribution of H1 sub-haplotypes which were compiled as combinations of the three SNPs. This analysis did not reveal any significant difference in sub-haplotypes involving SNPs rs242562 (G/A) and rs2435207 (G/A) between cases and controls (Table 5), as previously reported [16,30]. Finally, we estimated the effect of the rest two- and three-locus sub-haplotypes defined by SNPs rs242562, rs2435207 and rs 3785883, but the results showed no association with PD (data not shown).

**Table 2:** MAPT genotype frequencies in Parkinson's Disease.

	Genotype %				H1H1 vs. H1H2 and H2H2		
		
	H1H1	H1H2	H2H2	HWE P-value	OR	95% CI	*P *value
% cases (n = 122)	68.9	27.9	3.3	0.971	1.566	1.137–2.157	0.006
% controls (n = 123)	51.2	45.5	3.3	0.128	-	-	-

HWE, Hardy-Weinberg equilibrium; *P-*value, two-sided exact p-value from Fisher's exact test;

OR, odds ratio; 95% CI = 95% confidence interval.

**Table 3:** SNP1 and 2 genotype frequencies given as number (%).

	Cases (n = 84)			Controls (n = 63)			*P *value
rs242562*, 5' of exon 1							
Genotypic	G/G	G/A	A/A	G/G	G/A	A/A	0.806
	27(32.1)	45(53.6)	12(14.3)	23(36.5)	30(47.6)	10(15.9)	
Allelic	G	A		G	A		0.706
	99(58.9)	69(41.1)		76(60.3)	50(39.7)		

rs3785883, Intron 3							
Genotypic	G/G	G/A	A/A	G/G	G/A	A/A	0.388
	53(63.1)	29(34.5)	2(2.1)	35(55.5)	24(38.1)	4(6.4)	
Allelic	G	A		G	A		0.239
	135(80.4)	33(19.6)		94(74.6)	32(25.4)		

rs2435207, Intron 4							
Genotypic	G/G	G/A	A/A	G/G	G/A	A/A	0.832
	34(40.5)	41(48.8)	9(10.7)	28(44.5)	30(47.6)	5(7.9)	
Allelic	G	A		G	A		0.777
	109(64.9)	59(35.1)		86(68.3)	40(31.7)		

*P-*value, two-sided exact p-value from Fisher's exact test.

* rs242557 revealed identical genotypes in cases

**Table 4:** LD for H1-SNPs

	Cases (n = 84)		Controls (n = 63)	
	D'	R^2^	D'	R^2^
rs242562* vs rs3785883	0.136	0.007	0.005	0.000
rs242562* vs rs2435207	0.361	0.104	0.340	0.094
rs3785883 vs rs2435207	0.761	0.255	0.528	0.183

Linkage Disequilibrium (LD) measured by D' and *R*^2^.

* rs242557 revealed identical genotypes in cases

**Table 5:** Frequencies of sub-haplotypes (H1-SNPs rs242562-rs2435207) in study groups

Sub-haplotype	Cases Frequency	Controls Frequency	P-value	OR (95% CI)
G-G-H1	0.482	0.500	0.814	0.960 (0.737–1.250)
G-A-H1	0.107	0.103	1.000	1.025 (0.662–1.586)
A-G-H1	0.167	0.183	0.757	0.940 (0.671–1.316)
A-A-H1	0.244	0.214	0.579	1.103 (0.795–1.532)

*P-*value, two-sided exact p-value from Fisher's exact test; OR, odds ratio; 95% CI = 95% confidence interval.

## Discussion

Our results confirm that the distribution of the H1 haplotype of *MAPT *is an important risk factor of PD. Ôwo meta-analyses of studies on this subject supported the hypothesis that the H1 haplotype may confer susceptibility to PD,[18,38] and also recent PD genetic association studies with a large sample size observed a significantly greater frequency of the H1 haplotype in PD cases compared to control subjects [27,29,31,39]. Reasons for studying the relationship between H1 haplotype and PD were i) the proven increased transcriptional activity of H1 compared to H2,[20] suggesting a possible mechanism of PD susceptibility, since overexpression of transgenic tau has been found to cause neuronal death in Drosophila without neurofibrillary tangles,[40] and ii) a possible link between elevated H1 percentages among PD patients and tau mediated α-synuclein fibrillization,[6] which is believed to play an important role in the development and progression of PD. Our findings support this line of reasoning and provide further evidence for a role of this genetic variant of *MAPT *as a risk factor for PD.

Nevertheless, some conflicting findings have previously been related to varying effects of H1 in different ethnic populations [17,25]. Our study is the sixth conducted in a southern European population (3 Greek, 1 Italian, 1 Spanish, 1 Serbian) and the third in a Greek population. It is worth noting that five of these studies have also confirmed an association of haplotype H1 with PD while one Greek population study [17] failed to demonstrate this association. Generally, lack of reproducibility among case-control studies regarding the role of H1H1 genotype in PD can be explained by two factors. First, small sample sizes sometimes lead to underpowered studies. This is not always a satisfactory explanation since the association was not confirmed in a rather large group (n = 418) of German origin [25]. Moreover, the relevance of genetic risk factors could vary between different ethnic populations. The possibility of ethnic background influence among white Caucasians to explain the contradictory results has been discussed previously [25]. The genetic association between the overall *MAPT *locus and PD has been well established [18,31,41]. Other genes of the *MAPT *region either within the *MAPT *locus (e.g. *Saitohin*) or within the extended H1 haplotype (e.g.*KIAA1267*), have been also implicated in PD [31,42]. The genetic dissection of the *MAPT *locus and its role in PD is still a major challenge, because of the complexity of this genomic region. The latter is a result of both the complex haplotype structure and the fact that the *MAPT *locus contains other genes which are yet not fully characterized. Moreover, it is currently unclear whether the influence of these genes could vary among different ethnicities due to varying allele and haplotype frequencies within the *MAPT *locus. Our data contribute to the evaluation of *MAPT *in Greek patients among which the available studies revealed some contradictory results [16,17].

In the previous study in Greek patients a role of gender effects on the association between *MAPT *and PD was suggested [16]. The authors found in separate analysis according to sex a significant association between H1H1 and PD in men but not in women. This could point to a potential sex specific effect of genetic *MAPT *variation to PD susceptibility. However, it may be also related to a reduced statistical power in subgroup analysis. Hence, when we performed a separate statistical analysis for men and women in our cohort we also found a significant effect in 72 male patients but no significant effect in 50 female patients. In contrast, in logistic regression analysis we found no significant effect of sex status affecting the significant association between H1H1 genotype in the overall cohort. Our latter finding is in agreement with a large US case-control cohort study that did not support a sex specific effect [27].

Efforts were made in previous studies to analyze whether specific genetic variants in *MAPT *within the H1 haplotype are responsible for the increased PD risk. Risk variants on different H1 sub-haplotypes were found to contribute to population risk for PD. In each case the variant was found to be a different one: rs242562-rs2435207 G-A sub-haplotype in a Greek population,[16] A-A sub-haplotype for the same SNPs in a Norwegian population,[30] rs3785883 in another Greek population,[17] rs7521 and rs242557 in a Finnish population,[17] rs2471738 in a UK population [43] and finally a six-SNP sub-haplotype of H1 (hCV3202946, rs1800547, rs3785883, rs2435207, rs11568305, rs1078997) in a multicenter study conducted in Europe and North America [31]. On the other hand, Goris et al.,[29] and Winkler et al.,[25] did not find any association between PD and specific H1 sub-haplotypes. Taken together, published data failed to identify a common sub-haplotype within the H1 haplotype so far. In the case of sub-haplotypes analyses, the problem of statistical power might become even more important as the sample sizes are even smaller, since in some studies only the H1H1 carriers are examined. In this regard, we tried to add more data to the previous finding [16] in the Greek population by examining the same sub-haplotypes in the same ethnic background, but in patients obtained from a different site and geographic region. Our data reveal an almost identical percentage of rs242562 and rs2435207 genotypes and haplotypes among H1H1 patients and controls, showing that the risk does not seem to arise from one of the two previously reported sub-haplotypes composed by these SNPs. Furthermore, some of the sub-haplotypes defined by SNPs rs242562, rs2435207 rs3785883, which represented two and three-locus functional sub-haplotypes for the tauopathies,[35] do not seem to alter disease risk in Parkinson as it was also shown in other studies,[25,43] suggesting that Parkinson does not share the same pathways with the tauopathies.

A limitation of our study results from the small sample size and limited statistical power. As a consequence we examined, however, the consistency of our results by genotyping the H1H2 subjects of our cohorts (data not shown), namely 118 patients and 119 control individuals as previously suggested [35]. In this analysis we still did not identify any effect of single SNPs or SNP sub-haplotypes. In addition, although we used a well established standardized clinical scoring system for PD diagnosis misclassification of PD patients by lack of histological confirmation represents a potential other limitation of our study. Nevertheless, recently it was shown [43] in pathologically confirmed PD cases that the association of *MAPT *with the disease is chiefly driven by the H1/H2 division alone, which is in agreement with our results.

Thus, in order to dissect the genetic and molecular basis of the H1 haplotype in PD, additional studies and in larger samples are necessary. Nonetheless, since *MAPT *gene alters PD risk, identification of gene-gene or gene-environment interactions, contributing to accelerated degeneration of the nigrostriatal dopaminergic neurons, should be explored. Such knowledge about the underlying mechanisms could open up new arenas for early diagnostic and therapeutic interventions in this important neurodegenerative disease [44,45].

## Conclusion

Our data show strong evidence of an association between the H1/H1 genotype and PD in Greek population, however the SNPs rs242562, rs2435207 and rs3785883 within the H1 haplotype do not seem to alter susceptibility for PD.

## Competing interests

The authors declare that they have no competing interests.

## Authors' contributions

NR collected the samples, executed the genetic studies, performed the statistical analysis, interpreted the data and wrote the first draft of the manuscript. JB participated in the design and organisation of the project, helped to execute the genetic studies, helped to analyse the data and participated in the review and critique of the manuscript. GT carried out the examination of the patients and the collection of the samples and helped to review the manuscript. AO helped to analyse the data and participated in the critique of the manuscript. ND participated in the design of the project, helped to collect the samples, worked on data analysis and helped to draft the manuscript. RK developed the idea of the research initially, supervised the project and critically revised all the submitted material. All authors read and approved the final manuscript.

## Pre-publication history

The pre-publication history for this paper can be accessed here:


